# Mortality during Transport of Pigs Subjected to Long Journeys: A Study in a Large European Abattoir

**DOI:** 10.3390/vetsci9110590

**Published:** 2022-10-27

**Authors:** Eleonora Marti, Eleonora Nannoni, Giulio Visentin, Luca Sardi, Giovanna Martelli, Simona Belperio, Gaetano Liuzzo

**Affiliations:** 1 Department of Veterinary Medical Sciences (DIMEVET), University of Bologna, Via Tolara di Sopra 50, 40064 Ozzano Emilia, Italy; 2 Department of Public Health—Veterinary Service, Local Health Authority of Modena, Strada Martiniana 21, 41126 Modena, Italy

**Keywords:** pig welfare, road transport, long journeys, mortality, trailers, dead-on-arrival

## Abstract

**Simple Summary:**

Animal welfare during transport is a topical issue, also considering that a large number of animals intended for farming and slaughter are transported every day along roads and highways throughout Europe to meet market needs. Besides the well-known animal welfare concerns, conditions encountered during transport also have considerable consequences on the industry’s profitability. Few studies have analysed the effects of long journeys on both animal welfare and economic losses. The present study aimed to propose the use of transport mortality (dead-on-arrival animals, or DOAs) and data collected routinely by the slaughterhouses and the Public Veterinary Services during inspections as a simple and systematic screening method for identifying problematic journeys or transport conditions. These data allowed us to observe a similar mortality rate compared to other studies carried out in Europe (0.09%) and to identify a significant increase in mortality during the hot season and when prolonged stops (>60 min.) were carried out during long journeys. Based on this case study, routinely collected data may therefore be used as a simple screening method for identifying problematic journeys or transport conditions.

**Abstract:**

To date, especially in Europe, few studies have analysed the implications of long journeys on pig welfare and economic losses, expressed in terms of transport mortality. This study retrospectively analysed data collected from slaughtering registers and travel journals in a large Italian abattoir. We focused on pig transports coming from abroad and arriving at the slaughter plant after long journeys (a total of 59,982 pigs over 370 journeys). We explored the relationship between mortality and the following variables: country of origin, journey duration, astronomical season, stocking density on the truck, number of stops, and prolonged stops during the journey (lasting more than 60 min, likely due to traffic jams or truck problems). Overall, the low mortality rate observed (0.09%) was in line with European estimates. The factors with a significant or tendential effect on mortality during transport were the astronomical season (*p* = 0.0472, with higher mortality in spring) and the presence of prolonged stops during the journey (*p* = 0.069, tendential effect). Journey duration, stocking density, country of origin, and the number of stops were not statistically significant. In conclusion, based on this case study, using transport mortality combined with data collected during the common routine activity by the Public Veterinary Services in slaughterhouses could be a simple screening method for identifying problematic journeys or transport conditions.

## 1. Introduction

Animal welfare during transport is a topical issue since a large number of animals intended for farming and slaughter are transported every day along roads and highways throughout Europe to meet market needs. These animals are subjected in part to long journeys across countries.

The European legislation on the protection of animals during transport (Regulation 1/2005) [1] establishes differentiated legal requirements to be adopted based on the duration of the journey, classifying transport as short journeys (less than 8 h) and long journeys (more than 8 h). Notably, individual countries may have additional national requirements (e.g., travel duration or rest intervals). They may also have larger differences in road conditions, types of vehicles, animal genetics, and climatic conditions [2]. A recent recommendation [3] acknowledges the poor enforcement of Regulation 1/2005 and the lack of scientific literature on animal welfare during transport.

Among the available studies, environmental temperature and extreme weather are two of the most investigated factors. In particular, during the summer season, the mortality rate shows a significant increase [4]. Additional variables that affect animal welfare are conditions of animals at loading, mixing of groups, stocking density on the truck, duration of the journey, type of vehicle, vibrations on the vehicle, and animal handling practices [5]. Overall, the complex interactions between all these factors occurring during loading and transport complicate the identification of cause-effect relationships and the measurement of the magnitude of their effect on animal welfare [6,7]. For all these reasons, the welfare of animals subjected to long journeys should be carefully monitored. More space, lower temperatures, and shorter journeys were also recommended very recently by EFSA to improve animal welfare during transport [8].

Besides the well-known animal welfare concerns, conditions encountered during transport also have considerable consequences on the profitability for the industry. Animal losses can be defined as ‘pigs that die or become non-ambulatory at any stage of the marketing process, defined as movement from the grower–finisher environment to stunning at the abattoir’ [9]. They consequently determine economic losses due to carcass condemnation, carcass trimming, labour needed on the carcasses, and carcass disposal costs [10]. Of course, it should also be considered that death is the end of a long period of distress, and assessing mortality at unloading does not include all the other distressed or exhausted animals that survived the transport. Besides their evident poor welfare state, these animals, especially after long transportation, may show poor meat quality, resulting in secondary economic losses. To date, only a few studies have analysed the effects of long journeys on both animal welfare and economic losses. Mortality during long journeys has been estimated between 0.07% [11] and 0.11% [12]. According to Averós et al. [12], the mortality risk increased as the average time taken to load pigs decreased, when pigs travelled without fasting, or as a result of injuries, while other factors (country, loading density, water availability, and ventilation system) did not affect mortality. Regarding economic losses, high temperatures during transport were associated with lower meat quality in heavy pigs [13]; moreover, Nannoni et al. [10] observed that during summer the number of trucks having at least one dead animal during transport was 1.32 times more frequent compared to the winter season. Čobanović et al. [14] reported that both winter and summer resulted in the worst economic outcomes. During winter, they observed the highest prevalence of carcass lesions and red, soft, and exudative meat, while summer showed the lowest live and carcass weight, backfat thickness, pH, and prevalence of pale, soft, and exudative meat.

The aim of the present study was to propose transport mortality (dead-on-arrival animals or DOAs) after long journeys as a simple and systematic screening method for identifying problematic journeys or transport conditions. In this study, we used data collected during the common routine activity by the Public Veterinary Services in a slaughterhouse to calculate transport mortality and to identify sources of variation for pigs’ mortality recorded upon arrival at the slaughterhouse after long journeys.

## 2. Materials and Methods

### 2.1. Data Collection

Data collection was carried out in a commercial pig slaughterhouse located in the province of Modena, Italy. Approximately 1.3% of the batches (1.8% of pigs) which arrive at the slaughterhouse originate from European Union countries, and their meat is sold as fresh pork. This work focused on market-weight pigs (110–120 kg BW) that arrived at the slaughterhouse after long journeys from abroad between January 2018 and February 2021. During the unloading of the animals, the Public Veterinary Service carried out the *antemortem* examination and verified that the animals had been transported in compliance with the legal provisions of Reg. EC 1/2005 [1].

Our data collection was carried out retrospectively using slaughtering registers and travel journals provided by the Public Veterinary Service of the Local Health Unit of Modena, which operates within the slaughterhouse as the Competent Authority for official controls. Data was collated in an Excel dataset for the subsequent statistical analysis.

For each of the international journeys, the following information was collected from the slaughterhouse register and the travel journals:

- Date and season (astronomical season: spring from 21 March to 21 June; summer from 22 June to 22 September; autumn from 23 September to 21 December; winter from 22 December to 20 March);
- Number of pigs on the truck;
- Country of departure;
- Journey duration, min (calculated from the journey log);
- Batch weight, kg (truck weight difference before and after unloading);
- Available surface on the truck, m^2^ (across all loading floors);
- Stocking density on the truck, kg/m^2^ (calculated as batch weight/available surface);
- Number of stops during the journey (taken from the journey log);
- Duration of each stop, total and average duration of each of the stops, min;
- Mortality rate (% of animals transported);

The study design did not consider variables such as driver, type of vehicle, average temperature recorded during transport, density of the pigs in each compartment of the truck, and lairage duration, because we aimed to rely on data routinely collected during official inspections.

### 2.2. Statistical Analysis

Statistical analysis was conducted using SAS software (ver. 9.4; SAS Institute Inc., Cary, NC, USA). Factors associated with mortality rate were investigated through the Mixed procedure (PROC MIXED) with two different linear models. In the first instance, the linear model was as follows:

(1) $$y_{jklmno}=country_{j}+season_{k}+number\_stops_{l}+duration\_stop_{m}+loading\_density_{n}+e_{jklmno}$$

where $y_{jklmno}$ is the mortality rate, $country_{j}$ is the fixed effect of the *j*-th country of origin, $season_{k}$ is the fixed effect of the *k*-th astronomical season, $number\_stops_{l}$ is the fixed effect of the *l*-the number of stops, $duration\_stop_{m}$ is the fixed effect of the *m*-th stop duration, $loading\_density_{n}$ is the fixed effect of the *n*-th loading density, and $e_{jklmno}$ is the random residual $∼N\left(0,\;\sigma_{e}^{2}\right)$ where $\sigma_{e}^{2}$ is the residual variance. In this instance, we expressed loading density also as animals/m^2^ (instead of kg/m^2^). The variable was then discarded in favour of kg/m^2^ due to: (i) lower AIC (Akaike Information Criterion) for kg/m^2^, indicating better model fitting; (ii) absence of differences in model results using the two different definitions of loading density, and (iii) direct comparability of kg/m^2^ with the legislation requirements (same unit of measurement). A second linear mixed model was employed by substituting the effect of country of origin with the effect of journey duration; all the other fixed effects were those indicated for the first linear model. Table 1 summarises all fixed effects previously mentioned and their levels/quartile intervals. The inclusion of both country of origin and journey duration in the linear model was avoided due to the strong collinearity between the two terms. Customized hypothesis tests between groups of fixed effect levels were estimated using the ESTIMATE statement of the PROC MIXED. The threshold for statistical significance was set at *p* < 0.05.

## 3. Results

### 3.1. Descriptive Analysis

In the study period, 59,982 pigs (370 batches) of foreign origin were slaughtered. A total of 56 pigs were found dead at unloading (0.09%).

Batches originated mainly from Spain (67%), followed by France (25%), Germany (5%), and lastly, the Netherlands (2%).

Regarding seasonal distribution, 168 journeys were undertaken in winter, 100 in autumn, 67 in spring, and 35 during summer.

The following characteristics summarize the long journeys arrived to the abattoir in the study period:

- Average duration of the journey was 1184 min (about 20 h, range: 10–34.5 h);
- On average, only one stop was made (range: 0–5 stops);
- Stops lasted on average 56 min, with peaks of 480 min (8 h);
- Average batch size (pigs loaded on a trailer) was 162 (range: 106–185);
- Average loading density was 220 kg/m^2^ (range: 151–303).

For travel durations exceeding 24 h and loading densities above 235 kg/m^2^, the Competent Authority contested the related sanction pursuant to Italian Legislative Decree 151/2007 [15].

### 3.2. First Statistical Modelling

The first exploratory linear model included as variables the country of origin, the season, loading density, the number of stops during the journey, and the presence of long stops during the journey. Table 2 depicts the F-values and significance of fixed effects. The inclusion of the country effect in the model (France, Spain, Germany, and the Netherlands) was not statistically significant. The mortality rates for the four countries were 0.18, 0.17, 0.07, and 0.10, respectively (data not shown). A significant (*p* < 0.05) effect of the astronomical season was calculated, together with a tendential (*p* < 0.10) effect of long stops during the journey. Considering that journey duration was likely to be a better predictor of mortality during transport than the country of origin, the latter variable was replaced in the second modelling.

### 3.3. Second Statistical Modelling

In this model, the variable country of origin was excluded and replaced with the duration of the trip. Results, including estimates for each quartile/class, are shown in Table 3. Table 4 shows orthogonal contrasts that were carried out by grouping some classes to better understand their effect on mortality.

The second modelling confirms the significant effect of the season of the year (*p* < 0.05) and the tendential effect of long stops during transport (*p* < 0.10). Journey duration, loading density, and number of stops did not significantly affect transport mortality. Estimates highlighted higher mortality during spring and summer than in autumn and winter, with spring having the highest and winter having the lowest mortality. Lastly, trips that had at least one stop lasting more than 60 min showed tendentially higher mortality (*p* < 0.10) than those that took place with only shorter stops.

Orthogonal contrast between the hot seasons and the cold seasons shows that the hotter seasons increased mortality during transport by 0.18% compared to the colder seasons (*p* < 0.05, with a mortality in autumn-winter of 0.10%). Orthogonal contrasts between the presence and absence of stops showed a tendential effect, and stops increased mortality by 0.33% (*p* < 0.1, with a mortality of 0.065% in the absence of stops).

Journey duration and loading density did not show significant effects; however, numerically higher mortality (*p* > 0.1) was observed in Q2 (16.7–20.5 h) than in Q1 (lower travel duration), Q3, and Q4 (higher travel duration). For loading density, it can be observed that mortality numerically decreased from Q1 to Q3 as the loading density increased (*p* > 0.1). Unexpectedly, even in the NC class (journeys exceeding the EU legislation limit of 235 kg/m^2^) we observed extremely low mortality. However, this class included only 5 journeys carried out with very variable densities (236 kg/m^2^ to 302.9 kg/m^2^; data not shown).

## 4. Discussion

The mortality during transport obtained in this study (0.09%) is in line with what has been reported in the literature in recent years (0.07–0.09%) [11,12], although only a small number of scientific studies published assessed transport mortality during long journeys in Europe. According to older studies (1998), mortality during transport ranged from <0.1 to >1.0% percent in different European countries [16], and a comparable value (0.3%) was also reported by Corstiansen et al. in The Netherlands in 1977 [17]. It is likely that such a reduction over time might be due to the combined effects of genotype selection and progressively improved welfare conditions during transport, thanks to the evolution of the EU legislation and the means of transportation.

### 4.1. Effect of the Country of Origin

The lack of significant effects of the country of origin depends on the fact that for large countries, the nation itself is not necessarily associated with a longer or shorter trip duration. Knowing the geographical location of the farm of origin would allow a more precise estimate of the distance travelled, but this was not possible in the present study.

Furthermore, Regulation 1/2005 lays down specific requirements for transporter training and authorization, vehicle approval, animal handling and inspections, loading and unloading facilities, loading density, etc. (Chapter III) [1], resulting in quite uniform transport conditions which could explain the lack of significant effects of the country of origin.

### 4.2. Effect of Journey Duration

In the literature, both long and short trips are reported to be stressful for animals [5,18]. Generally, increased journey length is associated with increased transport losses [19]. On the contrary, in the present study, the overall effect of journey duration was not significant. However, there is a paucity of studies investigating the specific effects of journey duration separately from other factors [20], particularly in the framework of long transportation within the European context. To our knowledge, no study has systematically analyzed mortality as long journey duration progressively increased (i.e., from 8 to 24 h). On the other hand, the higher requirements for long journeys detailed in the Regulation should result in better comfort during transport, so that the effect of travel duration might not be as straightforward as expected. In fact, for long journeys, detailed provisions are also made for intervals for water and feed provision, the presence of ventilation systems and partitions within the truck, a roof of a light color, bedding provision, along with the need for a navigation system, a temperature monitoring system, and a journey log (Chapter VI of the Regulation) [1]. Concerning loading density, no specific provision is made for long journeys. However, the Regulation acknowledges that the minimum required surface area (maximum 235 kg/m^2^ for pigs of around 100 kg) has to be increased up to an extra 20% depending on the meteorological conditions, the journey time, and the breed, size and physical condition of the pigs [1]. With this respect, Gerritzen et al. [21] observed that pigs are more capable of adapting to long (550 km) transport conditions when loaded at a density below the present EU requirement.

### 4.3. Effect of Season

Our results with respect to season are confirmed by the scientific literature, which reports greater pig losses in summer [10,14,22,23]. Since pigs have poor thermoregulation abilities through their skin, temperatures above 20 °C are challenging for their ability to cope [24] and increase the pig mortality rate during transport [4]. Notably, in Europe, such temperatures can already be reached and exceeded in spring, although some studies highlit a limited increase in mortality at very high temperatures in North America [23]. It is worth highlighting that in our study, the number of trips recorded during summer was considerably lower than in other seasons (35 trips in summer, 67 in spring, 100 in autumn, and 187 in winter); therefore, mortality during summer may not be fully representative of the actual trends. This shortage of journeys during summer can be explained by the reduced demand for pork during this season in Italy [25].

### 4.4. Effect of Loading Density

The effect of loading density on the mortality rate was not significant. However, it should be highlighted that most authors found a positive correlation between stocking density and animal losses [26,27,28,29]. On the contrary, a decrease in mortality was found as loading densities increased in a study carried out at the same slaughter plant on a large population of heavy pigs [10]. Due to the scarcity of studies under European transport conditions, it would be more appropriate to collect data on a larger sample of long journeys to verify the real impact of different loading densities. The current legal provisions set a maximum limit for loading density (235 kg/m^2^) [1] but do not consider potential risks arising from low loading densities. For example, according to Barton Gade [18], at low loading densities, animals tend to walk and move around in the vehicle, exposing them to a greater risk of falls and injuries [18]. This may be attenuated by requiring the mandatory use of protections or internal partitions within the trailer for those transports that are carried out at low stocking densities.

### 4.5. Effect of the Number of Stops

Journeys with at least one stop showed tendentially higher mortality compared to journeys without stops (*p* < 0.10). The transport of pigs during long journeys can be conducted without breaks for a maximum of 24 h as long as animals have access to water [1]. However, the legislation relating to the transport of goods by road [30], to promote good working conditions for drivers and road safety, requires a mandatory 15-min stop at least every 4.5 h of driving, followed by a second stop of 30 min after another 4.5 h of driving. Therefore, as the duration of the trip increases, the number of stops necessarily grows, and it is impossible to evaluate the effects of the number of stops and the duration of the journey separately. Periods spent in a stationary trailer are known to be a high welfare risk, due to an uncontrolled increase in temperature and humidity inside the trailer compartments in the absence of natural ventilation, leading to increased transport losses [31]. In our study, the mortality during transport numerically increased (*p* > 0.10) as the number of stops increased. Although not significant, and also based on the previous literature, this seems to support the provisions of the EC Regulation 1/2005. Indeed, in addition using effective ventilation and water sprinkling in a stationary truck can reduce transport mortality [32,33] and improve pork quality [34].

### 4.6. Effect of Stops Duration

Stops lasting over 60 min tendentially increased transport mortality. These stops were recorded in 16 trips, and in most cases (13) they lasted over 120 min, reaching very prolonged durations (in 6 cases over 300 min, with peaks of 480 min). They were very likely due to problems with the vehicle or with road/traffic conditions that forcibly extended the duration of the journey, thereby exposing animals to unfavorable climatic conditions (both during summer and winter) and prolonged fasting. In particular, prolonged stops during the hot season expose animals to even more extreme climatic conditions and stress levels [5]. As mentioned above, in a stationary vehicle, the internal temperatures, in the absence of natural ventilation, can quickly reach high values, increasing transport losses, especially if no mitigating strategies are adopted (such as the fan-misting bank proposed by Pereira et al.) [31].

### 4.7. Limitations and Final Considerations

This study originated from the initial challenge to use only data routinely collected during official controls to identify problematic journeys. For this reason, some of the variables that are well known to have a crucial effect on animal welfare but are not officially inspected for each journey (environmental conditions and temperatures during transport and within the trailer, kind of trailer and type of transport, air conditioning, distance travelled, etc.) could not be used as explanatory variables. While it would be crucial in the future (also thanks to legislation improvements) to also routinely inspect temperature records within the truck, in this study we could only rely on the period of the year as an indicator of temperature.

## 5. Conclusions

The season of the year had a significant effect on transport mortality during long journeys (increased mortality in the hot seasons), while the presence of prolonged stops (likely related to issues during the journey) had only a tendential effect (increasing transport mortality by 0.33%). Loading density during transport and journey duration do not appear to be statistically significant hazards. However, due to the limited literature available to date on long journeys in the European context, and to the sometimes-conflicting results found in the literature with respect to these two parameters, it would be advisable to extend the study to a larger number of long journeys to draw stronger conclusions.

Based on this preliminary experience, using transport mortality combined with data collected during the common routine activity by the Public Veterinary Services in slaughterhouses could be a simple screening method for identifying problematic journeys or transport conditions. It would also be advisable that the assessment of transport outcomes in terms of mortality should be included in future legislation on the protection of pigs during transport.

## Figures and Tables

**Table 1:** Summary of the variables used for the statistical analysis and their classes or quartiles (Q).

Variable	N° of Levels	Classes/Quartiles
Country of origin	4	France Netherlands Germany Spain
Season	4	Spring Summer Autumn Winter
Number of stops	4	0 1 2 3–5
Loading density (kg/m^2^)	5	Q1: from 151.30 to 214.53 kg/m^2^; Q2: from 214.54 to 222.40 kg/m^2^; Q3: from 222.41 to 228.50 kg/m^2^; Q4: from 228.51 to 235 kg/m^2^; NC: non-compliant, i.e., above 235 kg/m^2^ (N = 5).
Long stops (>60 min)	2	NO; YES.
Journey duration (min)	4	Q1: from 600 to 995 min (from 10 to 16.6 h); Q2: from 996 to 1230 min (from 16.7 to 20.5 h); Q3: from 1231 to 1380 min (from 20.6 to 23 h); Q4: from 1381 to 2070 min; (from 23.1 to 34.5 h).

**Table 2:** Effects tested in the first modelling and their statistical significance. Significant effects are highlighted in bold, and tendential effects are underlined.

Effect	F Value	*p*-Value
Country of origin	0.45	0.7174
Season	3.49	**0.0159**
Loading density (kg/m^2^)	1.01	0.4035
Number of stops	0.80	0.4952
Long stops	3.68	0.0558

**Table 3:** Effects tested in the second modelling and their statistical significance (significant effects are highlighted in bold, and tendential effects are underlined. The table also shows the Least Square Means (LSM) estimates of transport mortality, Standard Error (SE), and significance for the different parameters considered in the statistical modelling. Significant effects are highlighted in bold, tendential effects are underlined.

Variable	Classes/Quartiles	LSM Estimates	Standard Error	F Value	*p*-Value
Journey duration (min)	Q1 Q2 Q3 Q4	0.1489 0.1883 0.1215 0.1371	0.05821 0.05407 0.05977 0.05806	0.65	0.5838
Season	Spring Summer Autumn Winter	0.2104 0.1818 0.1192 0.08439	0.05856 0.07307 0.05656 0.05145	2.67	**0.0472**
Number of stops	0 1 2 3–5	0.06544 0.1407 0.1932 0.1964	0.0706 0.05035 0.05214 0.08892	0.85	0.1995
Loading density (kg/m^2^)	Q1 Q2 Q3 Q4 NC	0.2165 0.1522 0.1491 0.1885 0.03841	0.04577 0.05158 0.05261 0.05085 0.1501	1.56	0.4920
Long stops	NO YES	0.08334 0.2145	0.03866 0.07621	3.43	0.0649

**Table 4:** Orthogonal estimates calculated for level groups of fixed effects (cold vs. hot season and stops during the journey, yes/no). Significant effects are highlighted in bold, tendential effects are underlined.

Variable	Classes	LSM Estimates	Standard Error	*p*-Value
Season	HOT (spring + summer) COLD (autumn + winter)	0.1886	0.08515	**0.0274**
Presence of stops	NO (0) YES (1–5)	−0.3340	0.1837	0.0698

## Data Availability

Data are available from the authors upon request.
